# A Rare Case of Typhoid Fever in the United States Associated With Travel to Mexico

**DOI:** 10.7759/cureus.22316

**Published:** 2022-02-17

**Authors:** Elina Kim, Jash Bansal

**Affiliations:** 1 Gastroenterology, Virginia Tech Carilion School of Medicine, Roanoke, USA; 2 Gastroenterology, Carilion Clinic, Roanoke, USA

**Keywords:** accurate diagnosis, adult hospital medicine, enteric fever (typhoid fever), travel health, travel vaccination, typhoid infection

## Abstract

Typhoid fever is an infectious febrile illness caused by *Salmonella typhi* that is rare in the United States but is endemic in regions of South Asia and Africa. Typhoid fever initially presents with nonspecific symptoms such as fever, malaise, and abdominal pain. We describe a case of typhoid fever in an adult in the United States with recent travel to Mexico. After a nonspecific presentation, the patient developed Faget sign and computed tomography (CT) of the abdomen revealed mesenteric adenitis, which prompted additional workup. Diagnosis of typhoid fever was established by blood culture and the patient was treated with ciprofloxacin.

## Introduction

Typhoid fever is a systemic illness caused by *Salmonella enterica* serotype Typhi (*S. *Typhi). The disease can initially present with nonspecific symptoms including low-grade fever and abdominal pain, making it diagnostically challenging for treating physicians. Typhoid fever can be deadly with its rapid progression to persistent high fevers and hemorrhagic shock within a few weeks. With timely treatment, mortality rates are 1-4%. Without appropriate therapy, mortality risk can be as high as 10-30% [1]. With more than 27 million cases and 200,000 deaths caused each year worldwide, typhoid fever remains a global health concern [2]. This is a growing problem because of the rise in drug-resistant strains. It is well known that typhoid fever is endemic in regions of South Asia and Africa. Typhoid fever is relatively rare in Latin America with the last major outbreak in Mexico being in the 1970s. However, in this study, we report a case of a 39-year-old male with typhoid fever in the United States with recent travel to Mexico. This case highlights the importance of a detailed history and physical, followed by a thorough diagnostic workup and the need to include typhoid fever as a differential diagnosis in a patient with recent travel history.

## Case presentation

A 39-year-old previously healthy male patient presented to the emergency department (ED) with a low-grade fever of 100 degrees F and nocturnal abdominal pain that would resolve by the morning and with ibuprofen. Associated symptoms included chills, nausea, constipation, decreased oral intake, and joint pain. The patient denied vomiting, hematochezia, melena, hematemesis, diarrhea, and rash. The patient had traveled to Mexico two weeks prior, where he consumed local food and water. He did not receive any traveler vaccinations or take prophylactic antibiotics. The patient denied any prior medical illness, hospitalizations, or surgeries. Family history was unremarkable. Mild elevations in aspartate aminotransferase (AST) of 84 U/L (normal: 8-33 U/L), alanine transaminase (ALT) of 95 U/L (normal: 7-55 U/L), and alkaline phosphatase of 168 IU/L (normal: 44-147 IU/L) were found. Complete blood count (CBC) and complete metabolic panel (CMP) were within normal limits. On this initial presentation, the patient was sent home on dicycloverine, which provided no relief.

As the days progressed, his symptoms continued and worsened to include symptoms during the day, prompting repeat presentation to the ED four days later. At that time, the patient’s vital signs included a fever of 103 degrees F, blood pressure of 90/58, and pulse of 83. On physical exam, the patient’s abdomen was soft with mild diffuse tenderness and normal bowel sounds without signs of peritonitis or appendicitis. CBC with differential and basic metabolic panel (BMP) were unremarkable (Table 1). Liver enzymes (AST, ALT, alkaline phosphatase) were persistently elevated (Table 1). Lactate dehydrogenase (LDH) was also elevated while lipase and erythrocyte sedimentation rate (ESR) were normal (Table 1). Calcium was incidentally found to be low (Table 1).

**Table 1:** Complete blood count, complete metabolic panel, ESR, LDH, and lipase levels ESR: erythrocyte sedimentation rate; LDH: lactate dehydrogenase; MCV: mean corpuscular volume; SEG: segmented neutrophils; AST: aspartate aminotransferase; ALT: alanine transaminase; CO2: carbon dioxide

Lab	Patient's Value	Reference Range & Units
WBC	4.1	4.0 - 10.5 K/uL
Hemoglobin	13.9	13.0 - 16.0 g/dL
Hematocrit	39.2	37 - 49%
MCV	89.9	78 - 98 fL
SEG	72.6	%
Lymphocytes	16.3	%
Platelets	145	130 - 400 K/uL
Sodium	139	135 – 145 MMOL/L
Potassium	3.7	3.5 – 5.3 MMOL/L
Chloride	102	95 – 107 MMOL/L
CO_2_	24	21 – 31 MMOL/L
Anion Gap	13	8 – 18 mmol/L
Urea Nitrogen	14	6 – 20 mg/dL
Creatinine	1.02	0.5 – 1.4 mg/dL
Calcium	7.5	8.5 – 10.7 mg/dL
Albumin	3.8	3.2 – 5.5 g/dL
Total Bilirubin	0.3	< 1.3 mg/dL
AST	69	8-33 U/L
ALT	87	7-55 U/L
Alkaline Phosphatase	165	44-147 IU/L
Lipase	57	13-60 U/L
ESR	34	0-22 mm/hr
LDH	394	140-280 U/L

**Figure 1:**
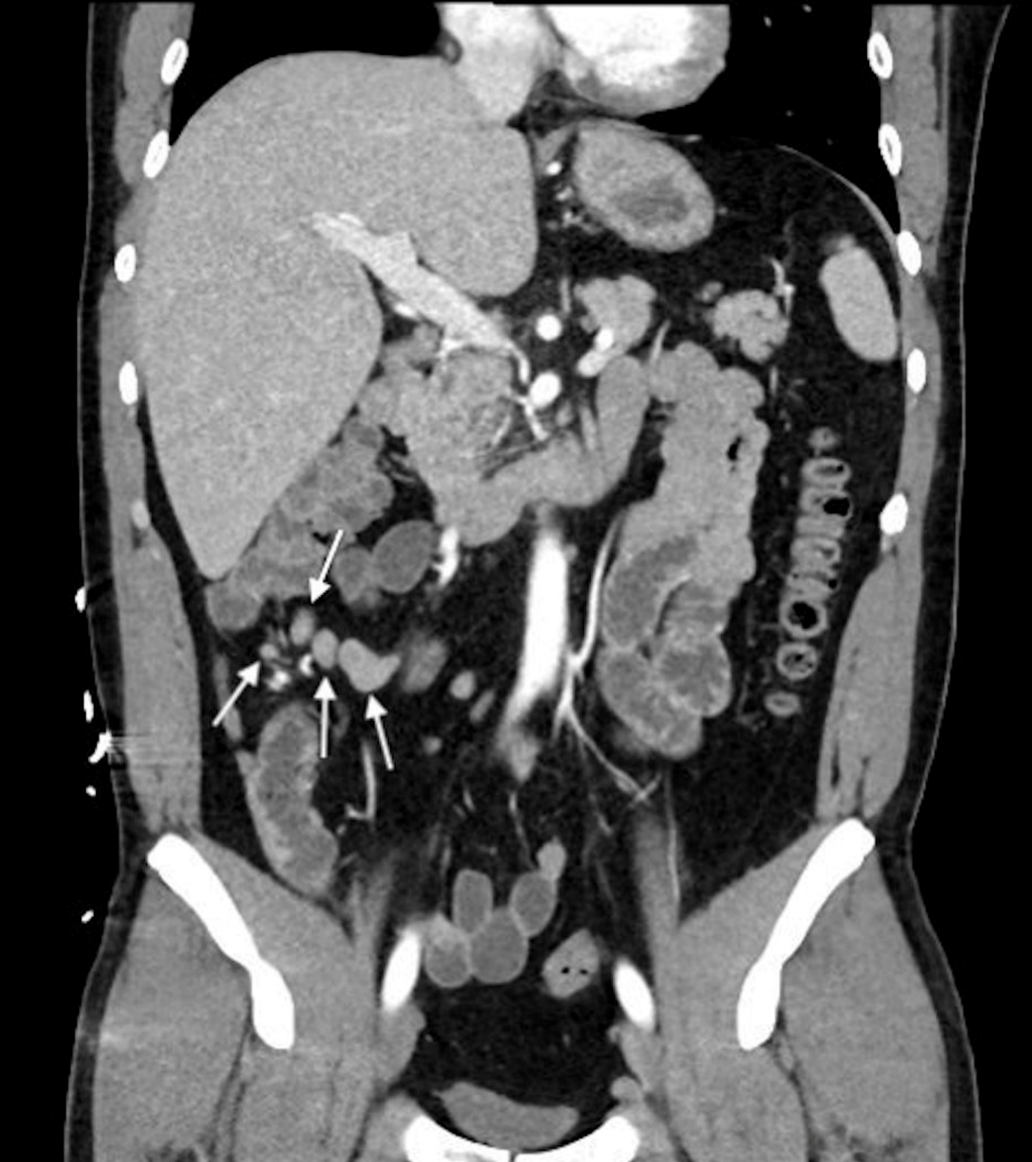
CT image of the abdomen with contrast revealing mesenteric adenitis (white arrows)

Computed tomography (CT) of the abdomen and pelvis with intravenous contrast demonstrated right lower quadrant mesenteric adenitis (Figure 1). Viral panels testing for Epstein-Barr virus (EBV), hepatitis A, hepatitis C, and human immunodeficiency virus (HIV) were all negative. Malaria smear and peripheral blood smear were unremarkable. No fecal WBCs were detected and Giardia-specific antigen was negative. Stool examination for ova and parasites revealed *Blastocystis hominis*. Blood cultures took three days to grow *S. *Typhi.

The patient was treated with ciprofloxacin and his fevers resolved within 12 hours. He was discharged the following day and ciprofloxacin was continued for a total of 14 days. Follow-up in the clinic one month after discharge was with complete resolution of symptoms. Repeat stool testing for chronic carrier state was advised, but the patient was lost to follow-up.

## Discussion

First described by Bretonneau and Louis in 1819, typhoid fever is a systemic illness caused by *S. *Typhi. There are historical descriptions of an infectious disease with similar characteristics dating back to 430 B.C. during the plague of Athens [3]. The infection is spread via fecal-oral contamination and is a more common problem where sanitation and sewage services are limited, such as in areas of South Asia and Africa [4]. Typhoid fever is relatively rare in Latin America with the last major outbreak in Mexico being in the 1970s. Although the incidence of typhoid fever in the United States has been low since the 1940s [4], it continues to be an important public health issue due to the high hospitalization rates, the spread of multi-drug resistant strains, and the increasing percentage of chronic asymptomatic carriers.

Clinical manifestations of typhoid fever are highly variable. They range from patients being asymptomatic to high fevers and hemorrhagic shock. Typically, the disease progresses in three stages. In the first week, symptoms include intermittent low-grade fevers, malaise, and changes in bowel habits. Signs may include relative bradycardia, also known as Faget sign, which is an exception to the traditional pattern where fever is accompanied by tachycardia. Faget sign is unique to certain infections including typhoid fever, yellow fever, brucellosis, Colorado tick fever, and some pneumonias (e.g. Legionella and Mycoplasma) [5], although it can also be seen with the use of beta-blockers [6]. Anemia and leukopenia or leukocytosis are also common findings during the first week of typhoid fever.

By the second week, the fever rises and becomes constant, around 104 degrees F (40 degrees C). Abdominal tenderness and hepatosplenomegaly may be present. As demonstrated in our patient, liver enzymes and serum C-reactive protein are frequently elevated. During the third week, severe complications can occur such as gastrointestinal perforation or hemorrhage due to sloughing and necrosis leading to bleeding from the terminal ileum, as well as encephalitis, respiratory distress, delirium, renal failure, and sepsis [7].

The relatively nonspecific signs and symptoms in the initial phase of typhoid fever make it a challenging diagnosis. Providers may mistake the nonspecific presentation for other common illnesses such as viral gastroenteritis or functional bowel disorders. Findings such as Faget sign in the absence of beta-blocker use and marked abdominal lymphadenopathy should prompt a more thorough laboratory workup. The diagnosis is often finalized by blood cultures, which are positive in 50-70% of cases [8]. Early in the course, stool culture is positive in 30-40% of cases. In our case, stool examination for ova and parasites revealed *Blastocystis hominis*, which is often associated with other infectious diseases. As typhoid fever progresses and patients develop systemic symptoms, bacterial levels in the colon decline and are rarely detectable [9]. CT scans are typically reserved for patients with complications of typhoid fever such as gastrointestinal perforation or bleeding. Findings on CT scans may include mesenteric lymphadenopathy, hepatosplenomegaly, gastrointestinal perforation, bleeding, abdominal abscess, and enteritis [10].

Typhoid fever is usually treated with fluoroquinolone, third-generation cephalosporin, or azithromycin.The antibiotic therapy should be based on culture sensitivity and if empirical, should be based on the drug resistance pattern in that area. The choice of antibiotic therapy is becoming increasingly challenging with the growing prevalence of antibiotic-resistant strains. In many regions in South Asia, over 80% of isolated *S. *Typhi are not susceptible to fluoroquinolones [11]. Rates of fluoroquinolone-resistant strains remain low in Africa but are rising [12]. Studies have also demonstrated a rise in strains resistant to ceftriaxone [13]. Typhoid fever has a mortality rate of 10-30% but is reduced to 1-4% with timely and appropriate antibiotic treatment [1].

The global annual incidence of typhoid fever is approximately 27 million cases, of which there are approximately 200,000 deaths [2]. Of these, only 200 to 300 cases occur within the United States [14]. About 80% of these cases occur among travelers to highly endemic regions, such as South Asia and Africa, while the rest are domestically acquired or have unknown travel history [15]. Despite the broad availability of vaccination, typhoid fever was the most common preventable disease among travelers in the United States with only 38% of patients traveling to endemic regions presenting for vaccination [16]. The CDC recommends vaccination in travelers to parts of the world where typhoid fever is common, individuals in close contact with a chronic carrier, and laboratory workers who work with *S.*Typhi.

## Conclusions

We describe a rare case of typhoid fever in the United States in an adult with recent travel to Mexico. This case highlights the importance of considering typhoid fever in the differential diagnosis for a patient with recent travel despite relatively nonspecific symptoms. In our case, the clinical suspicion increased in the setting of Faget sign and marked mesenteric lymphadenopathy. Typhoid fever is an important public health issue, and will only grow in significance with the increasing prevalence of multi-drug resistant strains. Patients traveling to endemic areas should be vaccinated.
